# Frontier research on mechanisms of bone destruction in rheumatoid arthritis

**DOI:** 10.3389/fcell.2026.1755990

**Published:** 2026-03-25

**Authors:** Hongting Lu, Niqin Xiao, Qianqian Yang, Yundong Xu, Jian Wang, Xi Liu, Qiumei He, Kuangmeng Chi, Yuanyuan Wei, Heguo Yan, Zhaohu Xie, Zhaofu Li

**Affiliations:** Yunnan University of Chinese Medicine, Kunming, Yunnan, China

**Keywords:** bone destruction, glycolysis, long noncoding RNA, programmed cell death, rheumatoid arthritis

## Abstract

Progressive bone destruction in rheumatoid arthritis (RA) represents a core pathological mechanism leading to joint dysfunction. Its pathogenesis involves multiple critical pathways, including abnormal activation of immune and synovial cells, excessive pro-inflammatory cytokine secretion, and disrupted bone remodeling balance. Recent advancements in research techniques and academic exploration have progressively refined our understanding of RA-related bone destruction regulatory mechanisms. Programmed cell death (PCD), long noncoding RNAs (lncRNAs), and the significantly enhanced glycolytic metabolism observed in RA patients all mediate the onset and progression of RA bone destruction by targeting these pathological pathways. These findings collectively reveal a complex regulatory network intertwining cell demise processes, epigenetic regulation, and metabolic reprogramming. This not only deepens our understanding of the pathological mechanisms underlying RA bone destruction but also provides novel theoretical support and potential therapeutic targets for developing multi-targeted intervention strategies against this pathological process.

## 1 Introduction

Rheumatoid arthritis (RA) is an autoimmune disease characterized by chronic, symmetrical polyarthritis with progressive bone destruction as its primary clinical manifestation. Under physiological conditions, bone remodeling is a dynamic process driven by osteoblast (OB) for bone formation and regulated by osteoclast (OC) for bone resorption to maintain equilibrium. These two cell types are crucial for dynamically regulating bone homeostasis, thus ensuring that the overall skeletal structure remains constant (Zhou et al., 2024; Modinger et al., 2018). In RA patients, bone erosion driven by OC-mediated bone resorption is predominant, with destruction of the bone surface impairing normal joint function and leading to structural deformities (Takeuchi et al., 2021). Approximately 90% of RA cases with persistent inflammation will develop clinical functional impairment within 20 years (Brown et al., 2017). The following are risk factors for bone destruction: female gender, early disease onset, prolonged disease duration, high-titer positivity for rheumatoid factor (RF) and anti-cyclic citrullinated peptide (CCP) antibodies, and elevated levels of inflammatory markers such as erythrocyte sedimentation rate (ESR) and C-reactive protein (CRP) (Liu et al., 2022).

Effectively suppressing bone destruction and reducing disability rates are the major challenges in the diagnosis and treatment of RA. Therefore, exploring novel mechanisms of disease pathogenesis is of significant importance. In recent years, breakthroughs and advancements in fundamental research on programmed cell death (PCD), long noncoding RNAs (lncRNAs), and glycolysis have provided fresh perspectives on understanding RA-related bone destruction. These emerging perspectives collectively broaden our understanding of the complexity of RA bone destruction and reveal potential new multi-target intervention strategies. In this review, we focus on the latest research advances in RA bone destruction and summarize the existing and future therapeutic strategies targeting these pathways.

## 2 Bone destruction in RA

RA is an autoimmune-driven, cascade process characterized by imbalanced bone metabolism. The pathological features of RA-associated bone destruction include innate immune dysregulation, a dysregulated cytokine network, activation of OC and chondrocytes, and the imprinting effect of resident stromal cells (Goldring, 2003). OC differentiate from precursor cells of the monocyte–macrophage lineage and are multinucleated giant cells primarily responsible for bone destruction in RA. The differentiation and maturation of OC primarily depend on the interaction between the nuclear factor kappa B receptor activator (RANK) and its ligand (RANKL) (Goldring, 2003). Under physiological conditions, the primary source of RANKL is OB. Certain immune cells and fibroblast-like synoviocytes (FLS) also participate in RANKL secretion (Jung et al., 2014). Upon binding of RANK to RANKL on the surface of OC precursor cells (OCPCs), the OC differentiation process is initiated. However, in RA-affected synovium, RANKL-expressing FLS and T cells aggregate around OCPCs. Under the influence of numerous pro-inflammatory cytokines, they activate the nuclear factor kappa-light-chain-enhancer of activated B cell (NF-κB) pathway and induce excessive RANKL expression. This promotes massive OC differentiation and maturation, which disrupts the bone remodeling cycle and induces joint destruction (Tanaka and Ohira, 2018).

Furthermore, FLS are the key cellular components driving the transition of synovial tissue from a healthy to a pathological state (Zhang et al., 2024). Under physiological conditions, FLS maintain synovial structure by synthesizing matrix proteins such as collagen while secreting joint lubricants such as hyaluronic acid and lubricin. This process ensures lubrication and nutrient supply to the cartilage surface (Komatsu and Takayanagi, 2022). However, in the pathological state of RA, activated FLS exhibit potent immunomodulatory capabilities and invasiveness. They activate inflammatory pathways through interactions with other immune cells and proliferate extensively to form synovial hyperplasia, thereby mediating damage to articular cartilage and bone (Cheng et al., 2021). Tissue-destructive RA-FLS induce OC formation by expressing the receptor activator of RANKL and activating the downstream TRAF6-NF-κB/MAPK signaling pathway while inhibiting OB formation via the secretion of Wnt inhibitors (Goldring, 2003). Meanwhile, through cross-talk with activated immune cells such as T cells and B cells, RA-FLS secrete pro-inflammatory cytokines and chemokines, which in turn orchestrate the recruitment and activation of monocytes and lymphocytes into the synovial tissue; promote the proliferation, migration, and tube formation of vascular endothelial cell (EC); induce M1 macrophage polarization; disrupt the Treg/Th17 balance; and amplify local inflammatory responses by facilitating NETosis (Nygaard and Firestein, 2020; Yue et al., 2025). Epigenetic alterations in FLS cells of RA patients lead to the persistent upregulation of MMPs, which degrade collagen-rich structures within joint tissues. This process represents a key factor in the destruction of articular cartilage and non-osseous supporting structures (Nygaard and Firestein, 2020; Croft et al., 2024). Excessive bone resorption by OC, aberrant invasion of RA-FLS, neovascularization, and amplification of local inflammatory signals ultimately cause irreversible bone erosion and bone loss. Throughout the above pathological process, the cross-talk between immune inflammation and bone metabolism permeates the entire course, and each stage is synergistically driven by multiple factors, including epigenetic modifications, metabolic reprogramming, and PCD.

## 3 Cutting-edge mechanisms of bone destruction in RA

### 3.1 PCD

**FIGURE 1:**
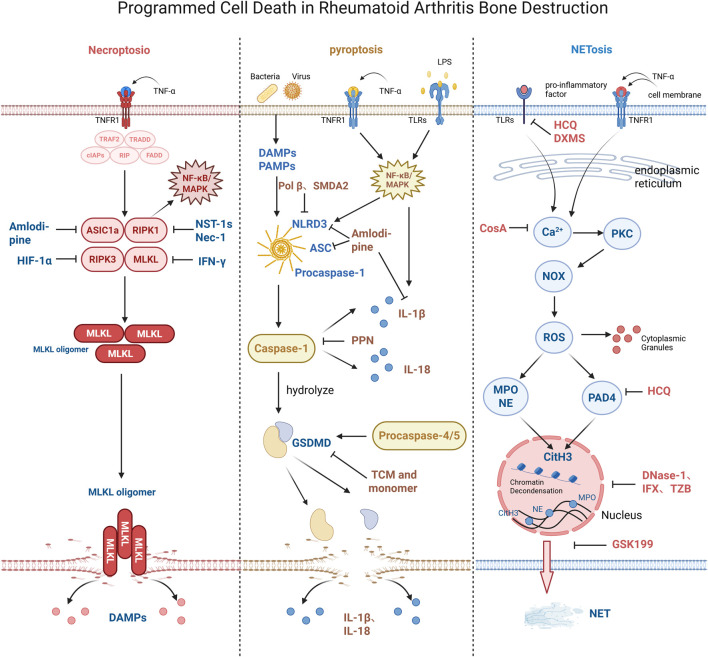
PCD in RA bone destruction. Necroptosis is induced by death receptor–ligand binding, which drives RIPK1/RIPK3 phosphorylation and necrosome formation. This activates MLKL, promotes its translocation to the plasma membrane, and causes membrane pore formation and cellular swelling, eventually leading to the release and inflammatory activation of DAMPs. NST-1s, Nec-1, IFN-γ, and HIF-1α inhibitors block necroptosis by inhibiting osteoclast activation and regulating the related cellular signaling pathways. Pyroptosis, an inflammatory programmed cell death, is initiated when the pattern recognition receptors sense signals and activate caspase-1/4/5/11. These caspases cleave GSDMD to generate N-terminal fragments that insert into the plasma membrane to form pores, causing cellular swelling, release of pro-inflammatory cytokines (e.g., IL-1β/IL-18), plasma membrane rupture, and robust inflammation. Amlodipine, Pol β, SMAD2, and active components of TCM suppress pyroptosis and exert osteoprotective effects in RA by inhibiting the overexpression of ASIC1a, ASC, NLRP3, and caspase-1 in RA-related cells. NETosis is a neutrophil death process triggered by pathogens or inflammatory factors, initiated via the NADPH oxidase-mediated reactive oxygen species pathway. It involves nuclear chromatin decondensation, nuclear membrane rupture, and chromatin fusion with cytoplasmic granular enzymes, ultimately leading to plasma membrane lysis and release of NETs. DNase-I, GSK199, IFX, TZB, CsA, and active components of TCM inhibit NETosis by cleaving DNA, suppressing release/formation of NETs, or reducing histone release, thereby abrogating its pathological role in RA-associated bone destruction. cIAPs, cellular inhibitor of apoptosis proteins; CitH3, citrullinated histone H3; FADD, Fas-associated death domain protein; LPS, lipopolysaccharide; MPO, myeloperoxidase; NE, neutrophil elastase; NOX, nicotinamide adenine dinucleotide phosphate oxidase; PKC, protein kinase C; PAD4, peptidyl arginine deiminase 4; ROS, reactive oxygen species; RIP, receptor-interacting protein; TRAF2, TNF receptor-associated factor 2; TRADD, tumor necrosis factor receptor type 1-associated death domain protein.

PCD is a genetically determined, active, and orderly mode of cell death (Nagata and Tanaka, 2017). By eliminating nonessential cells or cells undergoing specialization in the organism, PCD exerts a positive function in biological development, tissue homeostasis, and the response to external stimuli (Wallach and Kang, 2018). However, under specific pathological conditions such as RA, local PCD imbalance can induce cells to release immunostimulatory intracellular substances, trigger robust inflammatory immune responses, and mediate the pathological processes of synovitis and bone destruction in RA (Kolb et al., 2026) (see Figure 1).

#### 3.1.1 Necroptosis

##### 3.1.1.1 The role of necroptosis in RA bone destruction

As a novel form of cell death, necroptosis is distinctly separate from conventional apoptotic processes, and its regulation is independent of caspase activity (Ye et al., 2023). Necroptosis is triggered through the binding of specific death receptors (such as TNF receptor, Toll-like receptors, Fas ligand, and interferon receptors) to their corresponding ligands, initiating a complex cell death pathway. TNF-α stands as the most significant cytokine regulating this process in current research. By binding to TNF receptor 1 (TNFR1) on the cell membrane, TNF-α recruits downstream proteins to form complex I and modulates receptor-interacting protein kinase 1 (RIPK1). When caspase-8 is inhibited or knocked out, RIPK1 recruits RIPK3 to form necroptosomes, leading to RIPK3 phosphorylation and recruitment of mixed-lineage kinase domain-like pseudokinase (MLKL) to form complex IIb. Subsequently, MLKL is activated by phosphorylated RIPK3, forming a tetramer that inserts into the cell membrane, causing cellular swelling, membrane rupture, and the leakage of danger-associated molecular patterns (DAMPs) (Peng, 2024; Yang et al., 2022). In RA, DAMPs not only exacerbate the inflammatory response and enhance matrix metalloproteinases (MMPs) activity but also trigger chondrocyte release of pro-inflammatory cytokines, which causes persistent joint tissue damage. The intense inflammatory response induced thereby renders necroptotic cells significantly more immunogenic than apoptotic cells, leading to accelerated degradation of the extracellular matrix (ECM) (Khaleque et al., 2026).

Necroptosis represents a pivotal downstream target of inflammatory responses during RA progression (Rao et al., 2026). Significantly elevated expression of RIPK1, RIPK3, and MLKL is observable in peripheral blood mononuclear cells from RA patients, intestinal epithelium, and joint tissues of collagen-induced arthritis (CIA) mouse models. Concurrently, RIPK1 participates in the nuclear translocation process of NF-κB, a signaling pathway associated with OC generation (Raafat Ibrahim et al., 2022). The suppression of HIF-1α expression, a stabilizing inhibitor of RIPK3, inhibits necroptosis in intestinal epithelial cells and alleviates RA progression (Lyu et al., 2024). The necroptosis inhibitor necrostatin (NST)-1s suppresses OC activation and function by reducing the number of cells expressing OC markers tartrate-resistant acid phosphatase (TRAP), RANK, and RANKL in CIA mice while also modulating the Th17/Treg cell balance (Jhun et al., 2019). Conversely, interferon (IFN)-γ inhibits necroptosis by suppressing the production of cellular FLIP-like inhibitory protein (cFLIPL) and MLKL, thereby reducing interleukin (IL)-17, TNF-α, and Th17 cell expression, and delaying cartilage damage and joint inflammation in RA (Lee SH. et al., 2017).

##### 3.1.1.2 Therapeutic applications of necroptosis in RA

Macrophages in the synovial tissue of RA patients undergo necroptosis induced by TNF-α, accompanied by cellular structural damage. This leads to the secretion of the intracellular protein 14-3-3η into the serum and synovial fluid. Its expression levels correlate closely with disease activity and anti-citrullinated peptide antibody (ACPA) levels, making it a potential diagnostic marker for RA (Trimova et al., 2020). Acid-sensing ion channels (ASICs) are upregulated in the hypoxic microenvironment of RA. ASIC1a mediates necroptosis in AA rat articular cartilage damage via the RIP1/RIP3/p-MLKL pathway. Both the RIPK1 inhibitor necrostatin-1 (Nec-1) and the ASICs inhibitor amlodipine reduce TNF-α, IL-1β, and IL-6 levels. Both agents inhibit chondrocyte necroptosis via ASIC1a-mediated pathways and protect AA rat articular cartilage from damage (Chen et al., 2018). In CIA rats, the RIPK1 inhibitor KW2449 suppresses lipopolysaccharide-induced necroptosis while reducing joint swelling, bone destruction, tissue damage, and plasma inflammatory cytokine levels (Wang et al., 2023a). In RA, NE is activated by leukocyte differentiation antigen cluster 44 (CD44) and granulocyte-macrophage colony-stimulating factor (GM-CSF) to activate RIPK3 and MLKL. Studies have demonstrated that the application of fibroblast activation protein-alpha (FAP-α) and MLKL inhibitors effectively blocks the aforementioned joint enzyme-mediated necroptosis process, exhibiting significant potential in RA treatment (Wang X. et al., 2020). Irisin, a myogenic cytokine released by skeletal muscle and adipose tissue, exhibits significantly reduced peripheral blood levels in RA patients. Irisin exerts a marked inhibitory effect on TNF-α mRNA expression and modulates the necroptosis signaling pathway through its mitigating action on high-mobility group box 1 protein (HMGB1), which is a downstream target of necroptosis. It thus constitutes one of the protective factors against cartilage damage (Raafat Ibrahim et al., 2022).

#### 3.1.2 Pyroptosis

##### 3.1.2.1 The role of pyroptosis in RA bone destruction

Pyroptosis is a form of PCD identified and validated in recent years; it is a process dependent on the protease caspase-1 and the NOD-like receptor (NLR) and gasdermin (GSDM) protein families. The occurrence of pyroptosis primarily relies on two pathways, namely, the classical pathway dependent on caspase-1 and the noncanonical pathway dependent on caspases-4/5/11. Within the classical pathway, intracellular pattern recognition receptors (PRRs) recognize exogenous or endogenous signals, assembling inflammasomes to recruit and activate caspase-1. Both pathways subsequently cleave GSDMD *via* their respective activated caspases, causing oligomerization of the N-terminal pore-forming domain and the formation of large pores in the cell membrane. This process induces cell swelling, membrane rupture, and lysis, releasing pro-inflammatory factors such as IL-1β and IL-18 alongside other cellular contents. These signals propagate to neighboring cells, recruiting inflammatory cells and triggering tissue inflammatory responses—a feature that distinguishes it markedly from other cell-death patterns (Shi et al., 2017; Liu et al., 2024a; Anderson et al., 2023).

Inflammatory cytokines released via caspase-1-mediated pyroptosis promote RA progression by inducing inflammatory responses, immunocellular extravasation, vasodilation, and remodeling of adaptive immune responses (Fan et al., 2025). In synovial tissue cells from RA patients, the expression of NLRP3 (NOD-like receptor family, pyrin domain containing 3), caspase-1, and GSDMD is significantly upregulated (Wu T. et al., 2021; Zhang et al., 2023). Pyroptosis in RA-FLS, macrophages, chondrocytes, and OB plays a pivotal role in joint destruction and persistent inflammation. TNF-α, which is highly expressed under pathological conditions, acts as a key inducer of RA-FLS pyroptosis by regulating NLRP3 inflammasome activation and activating the NF-κB signaling pathway (Yu et al., 2021). Pyroptosis releases substantial pro-inflammatory mediators, including IL-6, IL-18, and IL-1β. This process is intrinsically linked to inflammasome involvement and positively correlates with inflammasome expression (Demarco et al., 2026). IL-1β activates RA-FLS and chondrocytes, inducing their production of MMPs and other inflammatory cytokines, thereby triggering bone matrix degradation and initiating/amplifying the inflammatory response. IL-18 promotes Th1 cell activation and proliferation, inducing IFN-γ secretion to enhance cellular immune responses; it also heightens the sensitivity of OCPC to RANKL, facilitating OC formation and activation, ultimately leading to bone erosion (Li et al., 2023). In RA patients, elevated expression levels of caspase-3 and GSDME-N in monocytes and macrophages, alongside TNF-α and complement C1q complexed with pentraxin 3(PTX3), can induce pyroptosis in these cells (Song et al., 2024). Similarly, within the hypoxic RA microenvironment, extracellular acidosis significantly upregulates pyroptosis-associated proteins such as ASIC1a, NLRP3, and caspase-1 in chondrocytes while simultaneously promoting the release of lactate dehydrogenase, IL-1β, and IL-18. NETs may also induce apoptosis in RA-FLS and promote phenotypic conversion by targeting the NF-κB/caspase 3/GSDME axis (Mao et al., 2024). These pyroptotic processes collectively mediate the inflammatory amplification and bone destruction progression observed in RA (Vasudevan et al., 2023).

##### 3.1.2.2 Therapeutic applications of pyroptosis in RA

Current research indicates that multiple drug components, bioactive molecules, and individual Chinese herbal constituents can counteract the progression of RA by inhibiting pyroptosis. ASIC1a inhibitors such as amiloride and amlodipine suppress the overexpression of ASIC1a, apoptosis-associated speck-like protein containing a CARD (ASC), NLRP3, and caspase-1 genes, as well as pro-inflammatory cytokines IL-1β and IL-18, in articular chondrocytes. This inhibits pyroptosis in chondrocytes, thereby exerting a chondroprotective effect (Zhou et al., 2025). DNA polymerase β (Pol β) is a key enzyme in base excision repair. DNA damage caused by Pol β deficiency activates the cyclic GMP–AMP synthase (cGAS)/stimulator of interferon genes (STING)/NF-κB signaling pathway, upregulating NLRP3, IL-1β, and IL-18 expression. This exacerbates macrophage pyroptosis and modulates the pathogenesis of RA. Promoting Pol β overexpression may represent an effective therapeutic target for preventing and treating RA and other related autoimmune diseases (Gu et al., 2022). SMAD2 (*Drosophila* mothers against decapentaplegic 2) is a protein involved in the transforming growth factor-β (TGF-β) signaling pathway. SMAD2 suppresses pyroptosis in RA-FLS by downregulating the NLRP3 inflammasome (NLRP3, ASC, and caspase-1 complex) and alleviates inflammatory cytokine secretion *via* the TGF-β signaling pathway, thereby improving RA symptoms (Mao et al., 2023). Periplogenin (PPN) is a natural cardiac glycoside that improves inflammatory responses in RA-FLS by inhibiting pyroptosis through regulation of the NLRP3/caspase-1/GSDMD signaling pathway, thereby reducing the release of inflammatory mediators (Ma et al., 2026). Quercetin, the most potent anti-RA active component in *Ligusticum chuanxiong*, acts as a natural caspase-1 inhibitor. By blocking caspase-1-mediated pyroptosis pathways, quercetin alleviates membrane disruption in RA-FLS cells (Ling et al., 2045). Gardenoside, the primary active component of the Chinese medicinal herb Gardenia, significantly suppressed the serum levels of TNF-α, IL-1β, and IL-18 in CIA rats. Concurrently, it downregulated NLRP3, caspase-1, and GSDMD expression in the synovial tissue, thus inhibiting macrophage pyroptosis (Fan et al., 2025). Phytosterols (Stig) alleviate RA progression by inhibiting Nrf2/NLRP3-mediated pyroptosis in chondrocytes (Ding et al., 2025). Baihu Guizhi Tang represents one of the classic traditional Chinese medicine (TCM) formulas for treating damp-heat patterns in RA. TLR4/PI3K/AKT/NFκB/NLRP3 pathway-induced pyroptosis may represent a viable target within the “immuno-inflammatory” imbalance network for reversing active RA progression via Baihu Guizhi Tang. Baicalin (MG) and chrysoberyl (CA) have been identified as representative monomeric constituents of TCM acting on this target (Li et al., 2022).

#### 3.1.3 NETosis and NETs

##### 3.1.3.1 The role of NETosis and NETs in RA bone destruction

NETs are fibrous networks protruding from the membranes of activated neutrophil (NE) and are composed of core components forming the main structure and granular components attached to the DNA backbone of the NETs (O'Neil et al., 2023). The structure of NETs enables them to function as both physical and antimicrobial barriers. At the sites of inflammation, they first restrict the extracellular spread of pathogens and subsequently degrade and kill them via NET-associated proteolytic enzymes, thereby exerting their effects across various pathological conditions (Akiyam et al., 2019). The release of NETs can be regarded as a specialized form of cell death, termed NETosis, providing dying neutrophils with a new pathway for sustained pathogen clearance. However, studies indicate that prolonged exposure to NETs-associated cascades correlates with autoimmune diseases and increases the risk of systemic organ damage. NETs accelerate inflammatory processes by releasing various bioactive molecules into the extracellular space, including DAMPs, histones, and active lysosomal enzymes (Lee KH. et al., 2017).

RNA sequencing of synovial fluid and synovial tissue from patients with severe RA revealed that NE in the synovial fluid promote inflammation by producing chemokines, ROS, and NETs (Wright et al., 2026). Schneider et al. (2024) demonstrated that NETs participate in the pathological process of RA bone destruction by regulating bone metabolic equilibrium and immune inflammatory responses. NETs significantly upregulate RANKL secretion by OB while suppressing osteoprotegerin (OPG) expression, leading to an abnormally elevated RANKL/OPG ratio (Zhao et al., 2024). This subsequently induces OC generation and activation, ultimately mediating RA joint destruction. The constituent elements of NETs (such as histones and extracellular DNA scaffolds) function as DAMPs, specifically recognized by pattern recognition receptors (PRRs) on OC precursor cell surfaces: Toll-like receptor 4 (TLR4) and TLR9. Activation of TLR4 and TLR9 initiates downstream cascade signaling through the NF-κB/MAPK pathways, driving the abnormal overexpression of the core transcription factor—the abnormal overexpression of activated T-cell nuclear factor c1 (NFATc1), which in turn significantly upregulates the transcription of OC-functional genes such as cathepsin K (CTSK), tartrate-resistant acid phosphatase (TRAP), and dendritic cell-specific transmembrane protein (DC-STAMP). This accelerates the differentiation and maturation of OCPC (O'Neil et al., 2023; Wu S. et al., 2021; O'Neil et al., 2022). Furthermore, NETs themselves can serve as a source of potential autoantigens. Compared with that in healthy controls (Khaleque et al., 2026) NETosis is enhanced in the serum and synovial fluid of RA patients. NETosis continuously exposes autoantigens—modified through processes like citrullination—to the immune system, thereby inducing the production of ACPA (Khandpur et al., 2013).

Additionally, identifying biomarkers in RA patients’ serum that enable early diagnosis and reflect inflammatory activity to assess disease activity has become a prominent research focus in recent years. The formation of NETs, particularly in the context of vascular involvement, may serve as a source of novel antigenic biomarkers for RA diagnosis (Trofimenko et al., 2021). NE biomarkers may more accurately reflect the disease status of RA patients than traditional inflammatory markers (such as ESR and CRP) (Frade-Sosa and Sanmarti, 2023). Detection of NET-derived products in peripheral blood, such as circulating cell-free DNA, free nucleosomes, and myeloperoxidase-DNA complexes, serves as an effective adjunct for the early identification of high-risk populations for RA. Free nucleosomes exhibit high specificity and positive rates in RA diagnosis and show significant correlations with disease activity and autoantibody positivity rates (Perez-S et al., 2017; Jariwala and Laxer, 2021).

##### 3.1.3.2 Therapeutic applications of NETosis and NETs in RA

Targeting the clearance of NETs and inhibiting their formation to alleviate the pathological progression of RA has become a current research focus. Numerous studies indicate that NETs inhibitors significantly reduce joint swelling, inflammatory responses, and structural damage. Deoxyribonuclease I (DNase-1) cleaves DNA *in vivo* and disrupts the backbone structure of NETs. Some scholars propose that elevated NETs levels in RA synovial fluid may stem from impaired DNase-1 activity or the presence of DNase-1 inhibitors, positioning DNase-1 as a potential therapeutic target for RA. Compared to healthy controls, RA patients exhibit significantly reduced serum DNase-1 activity, which correlates inversely with ESR, CRP, and NE counts (Xu et al., 2016). The HumDN1 VNTR allele, a gene associated with DNase-1 mRNA expression and enzyme activity, shows a significant association with RA susceptibility (AlFadhli and Ghanem, 2014). Additionally, after DNase treatment in antigen-induced arthritis (AIA) animal models, research workers observed a significant reduction in joint proteoglycan loss accompanied by a decrease in the number of OC around the joints (Schneider et al., 2024). DNase is currently utilized in the treatment of various diseases, including psoriasis, systemic lupus erythematosus (SLE), antiphospholipid syndrome, and malignant tumors. Leveraging DNase therapy’s short half-life characteristic, research workers have utilized hydrogels’ stability, controllable degradation, and protective properties for unstable drugs to prolong the retention of DNase coupled with oxidized dihydroxypropyl hyaluronic acid (OHA) within the joint cavity (Zhu et al., 2026). Regarding the inhibition of NETs formation, PAD4 inhibitors such as GSK199 can block the release of NETs, thus significantly reducing bone erosion in animal models. In clinical studies of disease-modifying antirheumatic drugs (DMARDs), a trial involving 106 RA patients showed that both the TNFα inhibitor infliximab (IFX) and the IL-6 receptor antagonist tocilizumab (TZB) significantly suppressed the formation of NETs. After 6 months of treatment, the inflammatory markers and pro-inflammatory factor expression decreased synchronously (Perez-S et al., 2017); cyclosporine A (CsA) inhibits the formation of NETs by suppressing the calcineurin pathway. Hydroxychloroquine (HCQ) may inhibit the production of NETs by regulating the interaction between MMPs and tissue inhibitors of metalloproteinases (TIMPs), thereby helping maintain extracellular matrix homeostasis (Mutua and Gershwin, 2021).

In addition, TCMs, herbal formulas, and natural products can exert therapeutic effects by modulating the balance of NETs formation. Natural compounds such as chuanxiong pyrazine, tetrahydrocoptisine, tanshinone IIA, emodin, and paeoniflorin polysaccharide alleviate NE-induced inflammation and oxidative stress, inhibit NE activation and DNA extrusion, block granule protein release, reduce histone release, and promote DNA degradation (Liu et al., 2024b; Lei et al., 2024). The blood-activating herb *Angelica sinensis* inhibits NE synthesis and the secretion of inflammatory cytokines and chemokines, reduces ROS production, and promotes NE apoptosis. The meridian-unblocking earthworm can suppress peripheral blood NETs release via the CXCL10/CXCR3 pathway (Homa, 2018); the Four Marvels Courage and Peace Decoction is commonly used to treat RA of the rheumatic fever type. It alleviates arthritis-related infiltration and cartilage damage by reducing the formation of NETs (Mutua and Gershwin, 2021). Cinnamon twig, peony root, and Anemarrhena decoction are commonly used to treat RA with mixed cold and heat patterns. It can alleviate RA by regulating the gut microbiota to reduce the formation of NETs (Ye et al., 2025).

### 3.2 LncRNAs

#### 3.2.1 The role of lncRNAs in RA bone destruction

LncRNAs are a class of RNAs longer than 200 nucleotides that lack protein-coding capability. They are primarily transcribed by RNA polymerase II and are widely distributed within the nucleus or cytoplasm (Wang P. et al., 2023). LncRNAs serve as crucial regulatory molecules involved in multiple vital processes, including chromosomal silencing, genomic imprinting, chromatin modification, transcriptional activation, transcriptional interference, and nuclear transport. Additionally, they can release microRNAs (miRNAs) from their translational suppression of the target messenger RNAs (mRNAs) by binding to them. By interacting with and modifying proteins, they regulate their activity, stability, and subcellular localization, thereby influencing downstream gene expression networks (Qiu and Chen, 2023; Grammatikakis and Lal, 2022). As key regulators of immune cell differentiation and activation, lncRNAs extensively participate in human disease processes by modulating gene transcription, regulating protein function, or acting as competitive endogenous RNAs (ceRNAs). Their role in autoimmune processes and autoimmune diseases has gained widespread recognition (Mizushima and Levine, 2020).

Dysfunction of lncRNAs is prevalent in RA patients and correlates with disease activity. Research workers identified 2,099 differentially expressed lncRNAs in peripheral blood mononuclear cells between RA patients and healthy controls, and 135 differentially expressed lncRNAs in knee synovial tissue. Furthermore, lncRNAs that are significantly upregulated in RA showed positive correlations with inflammatory markers such as serum CRP, IL-6, and TNFα, and the Simplified Disease Activity Index (SDAI) (Liang et al., 2019; Zhang et al., 2016). LncRNAs participate in multiple pathological processes of RA, including the positive or negative regulation of RA-associated inflammatory factors and immune cell gene expression through diverse molecular mechanisms; they influence synovial hyperplasia and contribute to articular cartilage damage and bone matrix destruction (Huang et al., 2021; Kolarz and Majdan, 2017). Through the application of differential expression analysis and comparative transcriptomics research methods, multiple LncRNAs with abnormal expression associated with RA bone destruction have been identified and validated, such as LncRNA ZFAS1, LncRNA GAPLINC, LncRNA SNHG14, and LncRNA PVT1 (Wang S. et al., 2020). In RA, FLS are one of the key pathogenic cells. The invasive and pro-inflammatory properties of RA-FLS are major drivers of synovial inflammation and articular cartilage damage in RA (Sun et al., 2026a). LncRNAs are key regulators of proliferation, invasion, migration, and autophagy in RA-FLS. LncRNAs function through miRNA-mediated regulation of target mRNA expression. For instance, compared to healthy controls, lncRNA ZFAS1 is significantly upregulated in the synovial tissue and RA-FLS from RA patients. ZFAS1 promotes RA-FLS invasion and migration through a miRNA-dependent mechanism by targeting and binding to miR-296-5p, miR-2682-5p, miR-27a, and other miRNAs, thereby reducing their levels (Ye et al., 2018; Zheng et al., 2021; Yang et al., 2026a). LncRNA GAPLINC may promote tumor-like proliferation and invasive behavior in RA-FLS via the miR-382-5p- and miR-575-dependent pathways while regulating pro-inflammatory factor secretion (Mo et al., 2025). In an arthritis mouse model, overexpression of LncRNA PVT1 significantly increased the levels of pro-inflammatory factors (TNF-α and IL-1β) secreted by RA-FLS while simultaneously suppressing the levels of anti-inflammatory factors such as IL-10 and IL-4 (Zhang et al., 2026). LncRNA SNHG14 targets macrophages as a key pathway, exerting dual pro-pathological effects: on the one hand, it promotes macrophage proliferation and M1 pro-inflammatory differentiation, increasing the number of inflammatory effector cells; on the other hand, it amplifies local inflammatory responses by enhancing the secretion of pro-inflammatory cytokines such as IL-6, IL-1β, and TNF-α, collectively mediating the formation of the inflammatory microenvironment and pathological damage in RA (Zhang et al., 2021). MMPs are key effector molecules in cell migration and invasion. On abnormal overexpression, the lncRNA NEAT1 promotes RA-FLS secretion of MMP-9 and TNFα, thus enhancing RA-FLS invasiveness while suppressing apoptosis. This ultimately exacerbates joint inflammation and tissue erosion in RA (see Table 1). Additionally, multiple lncRNAs have functions in the process of OC proliferation and maturation. Studies confirm the overexpression of lncRNA HOX in OC and bone marrow monocytes, whereas the lncRNA NTT/PBOV1 axis exerts a potential regulatory effect on monocyte differentiation in RA patients (Liang et al., 2019).

**TABLE 1:** The role of lncRNAs in RA bone destruction.

Cellular target	LncRNA	Full name	Expression in RA	Modulatory role in RA bone destruction	Target and pathway involvement	Significance	Reference
RA-FLS proliferation	LncRNA MGE3	Maternally expressed gene 3	Decrease	Reduced MGE3 levels increase the total distribution of RA-FLS cells in the S phase and G2/M phase, thereby promoting cell proliferation	LncRNA-MEG3/miR-93-5p/SMAD7 axis; reduced MEG3 expression diminishes the suppression of miR-93-5p, leading to targeted degradation of SMAD7 and resulting in abnormal activation of the TGF-β/SMAD pathway	MGE3 may serve as a diagnostic biomarker and potential therapeutic target	Liu et al. (2025), Tao et al. (2025)
	LncRNA HOTAIR	Hox transcript antisense RNA	Decrease	Reduced levels of HOTAIR promote the activation and proliferation of RA-FLS, leading to increased secretion of pro-inflammatory cytokines TNF-α, IL-6, and IL-1β, and matrix metalloproteinases MMP-1, MMP-3, and MMP-9	LncRNA HOTAIR/miR-106b-5p axis; reduced HOTAIR expression increases circulating miR-106b-5p levels, activating the NF-κB/MAPK and PI3K/AKT signaling pathways	HOTAIR levels are negatively correlated with RA disease activity measures (such as DAS28 scores, TNF-α, IL-6, and IL-1β levels)	Qiu et al. (2022), Liu et al. (2025a)
	LncRNA OIP5-AS1	Opa-interacting protein 5 antisense RNA 1	Increase	Elevated OIP5-AS1 levels upregulate the expression of genes involved in cell-cycle regulation, propelling RA-FLS from the G1 phase into the S phase and causing excessive proliferation of RA-FLS	LncRNA OIP5-AS1/miR-410-3p/Wnt7b axis activates the Wnt/β-catenin pathway; elevated OIP5-AS1 expression adsorbs miR-410-3p, thereby releasing its targeted inhibition of Wnt7b. This activation of the Wnt/β-catenin pathway promotes cell cycle progression, leading to abnormal proliferation of RA-FLS	​	Sun et al. (2023)
	LncRNA NFYB	Nuclear factor Y, beta subunit	Increase	NFYB upregulation promotes the elevated expression of cell proliferation-related target genes (Cyclin D1, c-Myc, and PCNA), causing an abnormally accelerated cell cycle progression in RA-FLS cells and significantly enhanced cell division and proliferation activity	LncRNA NFYB/ANXA2/ERK1/2 axis; elevated LncNFYB expression significantly upregulates ANXA2 protein levels, which in turn increases p-ERK1/2 protein levels. This drives the transcription and expression of downstream RA-FLS proliferation-associated target genes, exacerbating synovial tissue hyperplasia	NFYB may serve as a potential therapeutic target	Xiao et al. (2024)
	LncRNA RNA AL928768.3	​	Increase	Overexpression of RNA AL928768.3 promotes the upregulation of proliferation-associated genes, MMP-2, MMP-9, TNF-α, IL-1β, IL-6, and Bcl-2 while downregulating Bax and caspase-3 expression. This facilitates abnormal proliferation of RA-FLS, enhances their invasive and migratory capabilities, inhibits apoptosis, and amplifies local inflammatory signaling	LncRNA AL928768.3/LTβ/NF-κB axis; AL928768.3 directly binds and stabilizes the target protein LTβ (lymphotoxin β), inducing phosphorylation and degradation of the NF-κB pathway inhibitor IκBα. This subsequently promotes phosphorylation and activation of the p65 subunit, facilitating its nuclear translocation. This process initiates transcription of downstream proliferation-associated genes and pro-inflammatory genes	RNA AL928768.3 may serve as a diagnostic biomarker for disease, with its levels positively correlated with CRP and disease activity in RA patients	Sun et al. (2026c)
	lncRNA RP11-83J16.1	​	Increase	Elevated expression of RP11-83J16.1 enhances RA-FLS cell proliferation and invasive capacity and increases the levels of TNF-α, IL-1β, IL-6, MMP-3, and MMP-9	LncRNA RP11-83J16.1/URI1/β-catenin axis; high expression of lncRNA RP11-83J16.1 in RA significantly upregulates URI1 protein levels, leading to substantial cytoplasmic accumulation of β-catenin. This facilitates its binding with TCF/LEF transcription factors, thereby initiating the transcriptional expression of downstream proliferation-associated genes, invasion-related factors, and pro-inflammatory cytokines	RP11-83J16.1 may serve as a therapeutic target for disease treatment	Piao et al. (2021)
	LncRNA FOXD2-AS1	FOXD2 antisense RNA 1	Increase	FOXD2-AS1 overexpression drives the expression of key cell proliferation genes, accelerating the G1/S phase transition of the cell cycle, thereby causing abnormal activation and proliferation of RA-FLS	LncRNA FOXD2-AS1/miR-331-3p/PIAS3 axis; overexpression of lncRNA FOXD2-AS1 competitively binds and downregulates the protective factor miR-331-3p, leading to a significant upregulation of PIAS3 protein expression. This subsequently drives the transcription and expression of proliferation- and invasion-associated genes	​	Zhao et al. (2026)
RA-FLS anti-apoptotic	LncRNA GAS5	Growth arrest special 5	Decrease	Following GAS5 downregulation, expression of Bax, cleaved caspase-9, and cleaved caspase-3 was reduced, while Bcl-2 expression increased. This inhibited apoptosis in RA-FLS cells	LncRNA GAS5/miR-222-3p/Sirt1 axis; reduced GAS5 expression diminishes competitive binding to miR-222-3p, downregulates the protein expression of the protective mediator Sirt1, activates pro-inflammatory signaling pathways such as NF-κB, and inhibits apoptosis in RA-FLS	GAS5 may serve as a potential therapeutic target for RA treatment	Yang et al. (2026b)
	LncRNA NONHSAT042241	Non-host-associated transcript 042241	Decrease	Low expression of NONHSAT042241 downregulates Bax and cleaved caspase-3 expression, thereby inhibiting apoptosis and enhancing the invasive capacity of RA-FLS	LncRNA NONHSAT042241/Wnt/β-catenin axis; downregulation of NONHSAT042241 directly activates the canonical Wnt/β-catenin signaling pathway, promoting the expression of downstream proliferation-, inflammation-, and invasion-associated target genes	NONHSAT042241 may serve as a potential therapeutic target and is negatively correlated with RA bone destruction	Jin et al. (2024)
	LncRNA XIST	X inactive specific transcript	Increase	XIST upregulation reduced the expression levels of caspase-3 and Bax while elevating Bcl-2 expression, thereby inhibiting apoptosis in RA-FLS	LncRNA XIST/miR-126-3p axis; XIST acts as a negative regulator of miR-126-3p; miR-126-3p is an endogenous protective microRNA, whose downregulation inhibits apoptosis in RA-FLS and suppresses the activation of pro-inflammatory signaling pathways	​	Liu et al. (2025b)
T-cell activation and differentiation	lnRNA ITSN1-2	​	Increase	Elevated ITSN1 levels promote activation and proliferation of CD4^+^ T cells in peripheral blood, stimulate Th1/Th17 differentiation, and leads to increased levels of TNF-α, IL-6, MMP-1, and MMP-3	LncRNA ITSN1-2/miR-6823-3p/PELI3 axis; ITSN1-2 overexpression inhibits RIP2 K48-linked ubiquitination and degradation, leading to RIP2 protein accumulation and activation of the NF-κB/MAPK pathway	ITSN1-2 levels are positively correlated with disease activity markers (including DAS28-ESR, ESR, and CRP) and joint damage	Bian et al. (2025)
	LncRNA TUG1	Aurine upregulated gene 1	Decrease	In CD4^+^ T cells, reduced TUG1 levels increase the proportion of Th17 cells and expression of the characteristic transcription factor RORγt, promoting secretion of IL-17 and IL-23. Concurrently, it decreases the proportion of Treg cells and expression of the characteristic transcription factor Foxp3, inhibiting the secretion of IL-10 and TGF-β	LncRNA TUG1-BLIMP1-Th17/Treg axis; reduced TUG1 expression downregulates the levels of BLIMP1, a key transcriptional regulator of T cell subsets. In CD4^+^ T cells, this promotes Th17 cell differentiation while suppressing Treg cell function, leading to T cell subset imbalance and pro-inflammatory cytokine release. This indirectly regulates the activation and proliferation of RA-FLS and OC	​	Ye et al. (2024)
	LncRNA HOTTIP	HOXA transcript at the distal tip	Increase	HOTTIP promotes the regulation of CD4^+^ cell differentiation toward the Th17 subset and reduces the proportion of Treg cells, thereby causing an imbalance in the Th17/Treg cell ratio; it activates pro-inflammatory signaling pathways	LncRNA HOTTIP/miR-1908-5p/STAT3 axis; HOTTIP overexpression downregulates key transcriptional regulators in T cell subsets. In CD4^+^ T cells, it promotes Th17 cell differentiation while suppressing Treg cell function, leading to T cell subset imbalance and pro-inflammatory cytokine release	NEAT1 may serve as a potential diagnostic and therapeutic target	Yao et al. (2026)
Macrophage Activation and polarization	LncRNA H19	​	Increase	Elevated H19 levels promote M1 macrophage polarization; they increase the expression of TNF-α, IL-6, IL-1β, MMP3, MMP13, CCL8, CXCL9, CXCL10, and CXCL11	LncRNA H19/KDM6A axis H19 overexpression can upregulate KDM6A expression and activity, thus activating M1-type polarization genes	H19 levels were positively correlated with RA disease activity (DAS28 score), the proportion of M1-polarized macrophages, and the concentrations of TNF-α, IL-6, and IL-1β	Zhu et al. (2021)
	LncRNA MIAT	Myocardial infarction-associated transcript	Increase	MIAT suppresses macrophage hyperactivation; exerts endogenous anti-inflammatory effects; reduces secretion of TNF-α, IL-6, IL-1β, and IL-2; and inhibits pro-inflammatory signaling pathways such as NF-κB	LncRNA MIAT/miR-30a-5p/SOCS1 axis; MIAT significantly upregulates the protein expression level of SOCS1, a macrophage autophagy regulator, by binding to miR-30a-5p. This enhances macrophage autophagy activity, degrades inflammatory signaling molecules, and regulates inflammasomes	MIAT may serve as a potential therapeutic target for RA	Sun et al.a
Pro-inflammation pathway	lncRNA RNA143598	​	Increase	RNA143598 can amplify local inflammatory responses, thus elevating the levels of TNF-α, IL-6, IL-1β, and other cytokines; it activates the NF-κB signaling pathway, further promoting the transcription of downstream pro-inflammatory genes	LncRNA RNA143598/MTRNR2L1 axis; expression levels of RNA143598 and its transcribed gene MTRNR2L1 positively correlate with the infiltration of memory B cells, CD8^+^ T cells, and macrophages	RNA143598 serves as a diagnostic biomarker for RA, with its expression levels positively correlated with RF and anti-CCP antibody positivity rates	Wu et al. (2025)
	LncRNA NEAT1	Nuclear Enriched Abundant Transcript 1	Increase	NEAT1 amplifies local inflammatory responses by activating the pro-inflammatory NF-κB signaling pathway, leading to elevated levels of TNF-α, IL-1β, and IL-6	LncRNA NEAT1/miR-204-5p axis; elevated NEAT1 expression depletes miR-204-5p *via* its molecular sponge function, leading to reduced expression and functional suppression of this protective factor	NEAT1 may serve as a potential therapeutic target	Xiao et al. (2021)
	LncRNA SNHG14	Small nucleolar RNA host gene 14	Increase	Overexpression of SNHF14 amplifies local inflammatory responses, thus elevating the levels of TNF-α, IL-1β, IL-6, and other cytokines, while simultaneously mildly upregulating the expression of genes associated with cell proliferation	LncRNA SNHG14/miR-17-5p/MINK-JNK axis; SNHG14 overexpression downregulates the expression and activity of the protective factor miR-17-5p, significantly upregulating MINK1 protein expression levels, thereby inducing phosphorylation and activation of the JNK pathway	SNHF14 can serve as a diagnostic biomarker for disease, with its levels showing a positive correlation with CRP, ESR, and RF in RA patients	Zhang et al. (2021)
	LncRNA DSCR9	Own syndrome critical region 9	Decrease	Reduced DSCR9 levels promote inflammatory responses and hypercoagulable states in RA-FLS, leading to elevated levels of IL-6, IL-8, VEGF, and PAF in vascular endothelium and monocytes	LncRNA DSCR9/RPLP2/PI3K/AKT axis; DSCR9 inhibits NF-κB pathway activity by downregulating RPLP2 expression and suppressing PI3K/AKT pathway activation	DSCR9 levels were negatively correlated with clinical inflammation and coagulation markers but positively correlated with SF-36 scores	Wang and Liu (2024)
	LncRNA ITGB2-AS1, ICAM-1	Integrin subunit beta 2 -antisense RNA 1, intercellular adhesion molecules-1	Increase	Elevated levels of ITGB2-AS1 and ICAM-1 may enhance local inflammatory infiltration in RA, promoting the secretion of IL-6 and TNF-α; activates pro-inflammatory signaling pathways, thus accelerating OC activation	LncRNA ITGB2-AS1 and the ITGB2/ICAM-1 axis; ITGB2-AS1 enhances the stability of ITGB2 transcription and ITGB2 protein expression levels by complementary pairing with ITGB2 mRNA. The binding of ITGB2 to ICAM-1 activates the Wnt signaling pathway while simultaneously promoting intracellular tyrosine phosphorylation and Akt/ERK1/2 signaling activation	When used in combination for the clinical diagnosis of RA, both exhibit superior sensitivity and specificity compared to traditional biomarkers	Selim et al. (2025)

#### 3.2.2 Therapeutic applications of lncRNAs in RA

Increasing research evidence indicates that numerous abnormally expressed lncRNAs participate in the pathogenesis and progression of RA, potentially serving as therapeutic targets or biomarkers for this disease. Following RNA sequencing of serum exosomes from RA patients, research workers identified multiple lncRNAs, including TCONS_I2_00013502, ENST00000363624, ENST00000433825.1, and lnc-AL928768.3, that are specifically expressed in RA. This indicates these lncRNAs may serve as potential diagnostic markers for RA and are positively correlated with inflammatory markers such as CRP and disease activity (Wu et al., 2026; Sun et al., 2026b; Wen et al., 2020). Current drug development targeting lncRNAs has made progress, including the development of formulations such as small interfering RNAs (siRNA), miRNA, antisense oligonucleotides (ASO), and small-molecule inhibitors. Simultaneously, certain RA clinical therapeutic drugs have also been found to exert therapeutic effects by targeting specific lncRNAs (Huang et al., 2021).

For example, lncRNA-PICSAR may perform a key function in promoting synovial invasion and joint destruction by trapping miR-4701-5p in RA (Bi et al., 2019). The active component astragaloside in TCM can reverse the effects of lncRNAs overexpression on RA-FLS proliferation and cell cycle progression by interacting with the miR-17-5p/PDK1 axis via lncRNA LOC100912373 (Jiang et al., 2021). Plicarbin inhibits the secretion and expression of inflammatory cytokines such as IL-6 and IL-8 in RA-FLS by enhancing histone H3 acetylation at the promoter region of lncRNA NR024118 (Andújar et al., 2026). Tanshinone IIA promotes apoptosis in RA-FLS by upregulating the lncRNA GAS5 (Bioscience Reports, 2023). Research workers have developed a multifunctional drug-delivery system that co-delivers medications including methotrexate (MTX) and siRNA to local inflammatory sites. This approach demonstrates that MTX synergistically interacts with MMP-9 siRNA, exhibiting potent anti-inflammatory activity and reversing cartilage degradation (Yin et al., 2020). ASO also demonstrate significant potential in RA treatment. They can degrade lncRNAs or alter their function through various mechanisms (Alnefaie, 2020). Research has shown that ASO can regulate the progression of inflammatory diseases by targeting lncRNAs and inhibiting the infiltration of pro-inflammatory factors such as IL-6 and CXC chemokine ligand 16 (CXCL16) (Chen et al., 2024). In other autoimmune diseases, upregulation of lncRNA NEAT1 simultaneously promotes the expression of CXCL8 and TNF-α. Conversely, knocking down NEAT1 expression via ASO suppresses the expression of both CXCL8 and TNF-α in cells (Ye et al., 2026a). Japanese research workers demonstrated that ASO inhibit IL-1-stimulated proliferation of RA-FLS by analyzing its effects on mRNA and protein expression in synovial cells (Morita et al., 1997). In addition, considering the regulatory function of lncRNAs on mRNA, studies have utilized differentially expressed lncRNAs to evaluate the therapeutic response of RA patients to the TNF-α inhibitor etanercept (Wang et al., 2023c).

### 3.3 Glycolysis

#### 3.3.1 The role of glycolysis in RA bone destruction

RA is an autoimmune disease characterized by high metabolic demands. Mitochondrial respiration and glycolysis are the two primary pathways for energy production. Under oxygen-sufficient conditions, normal cells primarily generate ATP efficiently through mitochondrial oxidative phosphorylation (OXPHOS) (Angelin et al., 2017). However, RA synovial cells exhibit aerobic glycolysis—a phenomenon where cells preferentially utilize glycolysis under aerobic conditions and produce large amounts of lactic acid—also known as the Warburg effect (Johar et al., 2021). Aerobic glycolysis rapidly supplies biosynthetic substrates to pathological synovial cells and immune cells, directly participates in signal transduction, and drives cellular activation, proliferation, migration, invasion, and the production of inflammatory mediators (Gan et al., 2024). Enhanced glycolysis rapidly generates adenosine triphosphate (ATP), providing energy for OCPC differentiation. Simultaneously, the accumulation of lactate—a glycolytic metabolite—activates calcium–calmodulin kinase (CaN), upregulating the expression of the OC differentiation transcription factor NFATc1 (Zheng et al., 2024). Furthermore, lactic acid synergizes with hydrogen ions pumped out by the V-ATPase to acidify the resorption niche, thereby further promoting bone matrix degradation (Luo et al., 2023). The ATP production rate of glycolysis exceeds that of oxidative phosphorylation, rapidly supplying energy for RA-FLS cycle progression from the G1 to S phase and DNA replication. Inhibition of key glycolytic enzymes (such as PFKFB3) causes activation cycle arrest in RA-FLS cells, significantly reducing their proliferative capacity (Yi et al., 2022). The glycolytic key enzyme PFKFB3 metabolite fructose-2,6-bisphosphate (F-2,6-BP) regulates actin polymerization, enhancing pseudopod formation and migration capacity in RA-FLS; LDHA enhances RA-FLS invasion capacity by stabilizing integrin β1 function (Ye et al., 2026b). Furthermore, lactic acid has been recognized as a key mediator driving RA progression from early inflammation to late-stage bone destruction, and it also serves as a biomarker for RA progression. Lactic acid accumulated within the joint cavities of RA patients is transported via lactate transporters along concentration gradients to T cells, OCs, macrophages, and dendritic cells. By influencing the proliferation, polarization, differentiation, or phenotypic switching of these cells, it further promotes and sustains the progression of RA (Zheng et al., 2025; Yi et al., 2022).

**FIGURE 2:**
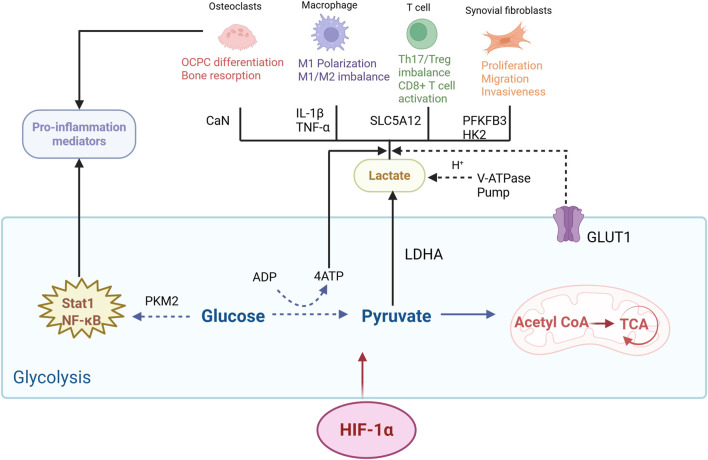
Glycolysis in RA bone destruction. In the local hypoxic microenvironment of RA, HIF-1α first upregulates the expression of glucose transporter GLUT1 to enhance cellular glucose uptake for glycolytic substrates, thereby driving the augmentation of aerobic glycolysis. This process modulates the activation and function of OC, RA-FLS, T cells, and M1 macrophages through multiple pathways and ultimately synergistically promotes the activation of bone-destructive cells, amplifies local inflammation, and accelerates the progression of RA-associated bone destruction. The specific mechanisms are as follows: ATP rapidly generated by glycolysis provides energy for the differentiation of OCs and RA-FLS; F-2,6-BP, a metabolite of the key enzyme PFKFB3, regulates actin polymerization to enhance the pseudopodia formation capacity of RA-FLS; HK-2 overexpression promotes the migration and invasion of RA-FLS via the FAK/ERK1/2/MMP-9 pathway; PKM2 induces the release of pro-inflammatory cytokines such as TNF-α and IL-1β through the Stat1 pathway; LDHA not only catalyzes the conversion of pyruvate to lactic acid but also elevates the invasive capacity of RA-FLS by stabilizing integrin β1. Lactic acid can activate CaN and upregulate NFATc1, and it synergizes with H+ to acidify the bone resorption microenvironment for OC activation; meanwhile, it promotes CD4^+^ T-cell retention and enhances fatty acid synthesis via SLC5A12 to supply energy for Th17 differentiation and strengthens TGF-β signaling through MOESIN-Kla modification to mediate Th17/Treg imbalance. In addition, lactic acid is taken up via MCT1 and converted to pyruvate by LDHB for the tricarboxylic acid cycle, which maintains mitochondrial function and ATP production in CD8^+^ T cells to enhance their survival. Furthermore, enhanced glycolysis leads to TCA cycle arrest and succinate accumulation, which further stabilizes HIF-1α and activates NF-κB, thereby promoting the expression of pro-inflammatory cytokines including IL-1β and TNF-α, along with M1 polarization markers. ADP, adenosine diphosphate; Acetyl CoA, acetyl-coenzyme A; CaN, calcium–calmodulin kinase; TCA, tricarboxylic acid cycle; SLC5A12, solute carrier family member 12.

Multiple proteins and enzymes involved in cellular glycolysis may represent potential therapeutic targets for RA. Glucose transporter 1 (GLUT1) mediates glucose transport across cell membranes and exhibits significantly elevated expression in the synovial tissue of RA patients. While facilitating glucose transport, GLUT1 stimulates the proliferation and functional effects of adaptive immune cells, along with the infiltration of pro-inflammatory factors. Intervening in GLUT1 expression *via* glycolysis inhibitors can improve joint inflammatory responses (Zezina et al., 2020) (see Figure 2).

#### 3.3.2 Therapeutic applications of glycolysis in RA

Hexokinases (HKs) are the key rate-limiting enzymes initiating glycolysis. Preclinical studies indicate that HK-2 overexpression promotes cell migration and invasion via the FAK/ERK1/2/MMP-9 pathway, whereas Hk2 knockdown inhibits the proliferation and invasion of RA-FLS through the glycolytic pathway (Garc et al., 2016). Moreover, the safety profile of HK2 as a therapeutic target stems from its global inhibition of glycolysis. 2-deoxy-D-glucose (2-DG) blocks glycolysis by inhibiting HK2, thereby reducing joint swelling, alleviating bone destruction, suppressing synovial cell proliferation and migration, and diminishing synovial cell secretory function in animal models (He et al., 2023; Wang et al., 2026); Phosphofructokinase (PFK) catalyzes the conversion of fructose-6-phosphate (F-6-P) to fructose-1,6-diphosphate (F-1,6-BP), thus serving as the second key rate-limiting enzyme in glycolysis. Previous studies indicate that PFK2 performs a pivotal function in the “Warburg effect.” The TCM formula Qingre Huoxue Tang may reduce disease activity in RA, mitigate inflammatory responses, and delay bone destruction by inhibiting F-1,6-BP 1 and activating the AMP-activated protein kinase (AMPK) signaling pathway (Zhang et al., 2025). Pyruvate kinase M2 (PKM2) is the rate-limiting enzyme catalyzing the final reaction of glycolysis. Experimental studies reveal that PKM2 is overexpressed in macrophages within the spleen and synovial tissues of rat models of arthritis. It promotes the production of pro-inflammatory factors such as TNFα and IL-β through the signal transducers and activators of transcription (Stat)1 signaling pathway, thereby inducing pro-inflammatory macrophage activation. Additionally, it mediates glycolytic reprogramming by activating the Akt/mTOR signaling pathway, thus contributing to the progression of RA (Xu et al., 2020). Inhibition of PKM2 significantly improves clinical symptoms in arthritic rats, reduces synovial hyperplasia and bone destruction, and suppresses RA-associated bone erosion. Therefore, PKM2 may serve as a candidate therapeutic target for RA (Liao et al., 2026). The active component icariin in TCM can reduce the pro-inflammatory M1 macrophage polarization by synergistically inhibiting macrophage glycolysis and regulating PKM2 reprogramming. This inhibits OC differentiation, thereby alleviating the pathological process of bone destruction in RA (Yan et al., 2024). Lactate dehydrogenase A (LDHA) reduces pyruvate to lactate during glycolysis. During OC differentiation, LDHA expression is upregulated alongside lactate transporter monocarboxylate transporter 4 (MCT4). LDHA inhibitors suppress OC differentiation and reduce their bone resorption activity, thereby mitigating excessive bone resorption in RA (Nishioku et al., 2023). Echinomycin, an inhibitor of hypoxia-inducible factor (HIF)-1α, not only suppresses the expression of GLUT1, LDHA, and MCT4 but also blocks OC differentiation and inhibits their bone resorption activity (Nishioku et al., 2025). Research utilizing ultrasound-targeted microbubble destruction (UTMD) has attempted to enhance mesenchymal stem cell (MSC) migration and improve cartilage repair efficiency by activating the HIF-1α-mediated glycolytic pathway, thereby playing a role in the treatment of OA (Kong et al., 2026). Additionally, retinoic acid receptor-related orphan receptor (ROR) α, a negative regulator of inflammatory responses, suppresses the expression of glycolysis-related genes in T cells within experimental RA mouse models. It directly influences the formation of OC, thereby mitigating progressive joint damage associated with RA (Park et al., 2019).

## 4 Conclusion

RA bone destruction is an autoimmune-driven cascade process involving bone metabolic imbalance. The interaction between epigenetic modifications mediating genetic susceptibility (e.g., susceptibility genes HLA-DRB1, PTPN22, and TRAF1-C5) and environmental triggers (e.g., smoking, periodontitis, and mechanical stress) constitutes the initial step in disease onset. Environmental factors alter epigenetic marks to regulate target gene transcription, thereby activating two key pathways, namely, self-antigen presentation and adaptive immune activation and RANKL/OPG axis imbalance and recruitment of OC precursor cells. The migration of RA-FLS precursor bone marrow mesenchymal stem cells to synovial tissue establishes the molecular basis for bone destruction. Persistent immune-inflammatory stimulation confers an invasive phenotype on RA-FLS, characterized by enhanced activation, antiapoptotic capacity, and invasive ability. This stage manifests as marked bone metabolism imbalance and massive immune cell infiltration. Invasive RA-FLS target monocytes and lymphocytes by releasing pro-inflammatory mediators and cytokines, promoting their recruitment and activation within synovial tissue. They also stimulate vascular EC proliferation, migration, and luminal formation; polarize M1 macrophages; disrupt the Th17/Th17 ratio; and amplify local inflammation through NETosis. More critically, RA-FLS highly express RANKL, which directly binds to RANK on OCPC, activating downstream TRAF6-NF-κB/MAPK pathways to promote OC differentiation, activation, and recruitment. Excessive bone resorption by OC, abnormal invasion by RA-FLS, angiogenesis, and amplified local inflammatory signals ultimately lead to irreversible bone erosion and bone loss. Throughout these pathological processes, the interplay between immune inflammation and bone remains constant, with each stage synergistically driven by multiple factors, including epigenetic modifications, metabolic reprogramming, and programmed cell death.

In this review, we primarily focus on the roles and therapeutic targets of three mechanisms—PCD, lncRNA, and glycolysis—that have gained significant research attention in recent years regarding their involvement in bone destruction in RA. PCD refers to the genetically regulated, autonomous, and orderly death of cells to maintain homeostasis. However, under RA pathological conditions, local PCD dysregulation may act as a pathological factor mediating the synovitis and bone destruction processes in RA. In this study, we systematically elucidate the core roles of three forms of programmed cell death—NETosis, necroptosis, and pyroptosis—in the bone destruction process of RA: NETosis mediates citrullinated protein production, triggering autoimmune responses and accelerating bone erosion; necroptosis induces the release of cellular contents, promoting inflammatory amplification and immune cell infiltration; pyroptosis activates inflammasomes by secreting pro-inflammatory factors such as IL-1β and IL-18, further disrupting OC function and enhancing RA-FLS invasiveness. LncRNAs act as epigenetic factors that influence the pathogenesis and progression of RA by regulating bone homeostasis-related cells and multiple signaling pathways, thus performing a crucial function in the pathological mechanisms of RA-related bone destruction. On the one hand, lncRNAs can directly activate the transcription of pro-inflammatory and pro-invasive mediators in FLS by modulating histone modifications and binding transcription factors. Alternatively, they may sequester miRNAs within FLS, thereby releasing their suppression of target genes, enhancing the activated phenotype of FLS, and promoting OC differentiation. As an autoimmune disease with high metabolic demands, synovial cells in RA exhibit a metabolic profile characterized by preferential glycolysis under aerobic conditions. OC differentiation and bone resorption processes are highly dependent on glycolysis. Glycolysis rapidly supplies biosynthetic substrates and energy to RA-FLS and immune cells, directly promoting synovial cell activation, proliferation, migration, invasion, and inflammatory cytokine secretion. Furthermore, the glycolytic metabolite lactate stimulates OC precursor macrophages toward pro-inflammatory M1-type differentiation and directly enhances OC-mediated bone resorption, serving as a core mediator in RA progression from early inflammation to late-stage bone destruction.

Although current research has achieved significant breakthroughs, certain limitations remain. For instance, the cross-regulatory networks among different types of PCD remain unclear. We need to integrate multiple PCD forms—including apoptosis, necroptosis, autophagy, ferroptosis, and pyroptosis—into a unified research framework to comprehensively examine their mutual influences and underlying mechanisms in RA-induced bone destruction. Regarding lncRNAs, challenges include the absence of standardized detection methods, poor reproducibility due to naturally low expression levels, and insufficient large-scale clinical validation. Solutions require establishing standardized detection systems, optimizing highly sensitive detection technologies, and clarifying their clinical value through integration with imaging characteristics. Regarding glycolysis, the key molecules regulating metabolic reprogramming in RA-related bone destruction remain unidentified. Solutions require focusing on screening and validating critical metabolic enzymes, thus optimizing targeted intervention strategies through integrated *in vitro*/*in vivo* experiments and preclinical studies. Future efforts should integrate multiple regulatory networks to elucidate their synergistic mechanisms in mediating RA bone destruction. Building upon this understanding, multi-target combined intervention strategies can be developed, providing experimental support and clinical translation evidence for precision prevention and treatment of RA bone destruction. This will lay a solid foundation for pioneering novel RA therapeutic pathways, thus enhancing treatment efficacy and improving the patients’ quality of life.

## Glossary

A**DAMTS9**: A disintegrin and metalloproteinase with thrombospondin type 9
AIA: Antigen-induced arthritis
ASO: Antisense oligonucleotides
AMPK: 5′-Adenosine monophosphate-activated protein kinase
CRP: C-reactive protein
CXCL: C–X–C motif chemokine ligand
CsA: Cyclosporine A
DAMPs: Damage-associated molecular patterns
DNase-1: Deoxyribonuclease 1
DMARDs: Disease-modifying antirheumatic drugs
ESR: Erythrocyte sedimentation rate
FLS: Fibroblast-like synoviocyte
F-6-P: Fructose-6-phosphate
F-1,6-BP: Fructose-1,6-diphosphate
GLUT1: Glucose transporter 1
HKs: Hexokinases
HCQ: Hydroxychloroquine
IFX: Infliximab
PI3K/AKT: Phosphoinositide 3-kinase/protein kinase B
JNK: c-Jun N-terminal kinase
LDH: Lactate dehydrogenase
LncRNA: Long noncoding RNA
MPO: Myeloperoxidase
MMP: Matrix metalloproteinase
miRNA: MicroRNA
mRNA: Messenger RNA
MINK1: Misshapen/nik-related kinase
MTX: Methotrexate
MDA5: Melanoma differentiation associated gene 5
MCT4: Monocarboxylate transporter 4
NETs: Neutrophil extracellular traps
NF-κB: Nuclear factor κ-light-chain enhancer of activated B cells
NO: Nitric oxide
OCPCs: Osteoclast precursor cells
OC: Osteoclast
OB: Osteoblast
OXPHOS: Oxidative phosphorylation
OCR: Oxygen consumption rate
OA: Osteoarthritis
OHA: Oxidized dihydroxypropyl hyaluronic acid
PAD: Peptidylarginine deiminases
p-IκBα: Phosphorylated nuclear factor of κ-light chain enhancer of activated B cells inhibitor alpha
P-P65: Nuclear factor kappa B subunit p65
PPR: Proton production rate
PFK: Phosphofructokinase
PKM2: Pyruvate kinase M2
RA: Rheumatoid arthritis
RF: Rheumatoid factor
RANK: Nuclear factor kappa B
RANKL: Nuclear factor kappa B ligand
ROS: Reactive oxygen species
ROR: Retinoic acid receptor-related orphan receptor
siRNA: Small interfering RNAs
Stat: Signal transducer and activator of transcription
SLE: Systemic lupus erythematosus
TNF-α: Tumor necrosis factor**-α**
TZB: Tocilizumab
TIMPs: Tissue inhibitor of metalloproteinase
UTMD: Ultrasound-targeted microbubble destruction
Wnt/β-catenin: Wingless/Int-β-catenin
YY1: Yin Yang 1
2-DG: 2-deoxy-D-glucose
